# Toward Better Teamwork: Recommendations for Improved Integration of Epidemiology and Risk Assessment

**DOI:** 10.1289/ehp.119-a525a

**Published:** 2011-12-01

**Authors:** Bob Weinhold

**Affiliations:** Bob Weinhold, MA, has covered environmental health issues for numerous outlets since 1996. He is a member of the Society of Environmental Journalists.

A four-person team of epidemiologists and risk assessors has taken a somewhat unusual step for their fields: working closely together. Concerned that their colleagues don’t collaborate much, despite the interdependence of their work and their mutually shared goal of improving public health, the team developed recommendations designed to alleviate this problem **[*EHP* 119(12):1671–1675; Fann et al.]**. The recommendations focus on air pollution but could be adjusted to work for many other topics, such as water pollution, food contamination, or exposure biomarkers.

The primary focus of their recommendations is the need for epidemiologists to report specific data elements that would help risk assessors avoid misinterpreting epidemiologic findings, more accurately quantify risks, and better advise policy makers. These additional data could also help epidemiologists who conduct meta-analyses. The team says adding this information requires only modest adjustments in typical practices.

One straightforward suggestion is to report detailed data on all confounders considered—in the case of air pollution studies, this might include other pollutant exposures, use of air conditioning, and demographic variables such as age, sex, race, income, and education. Researchers should also specify diagnostic codes used, list the data source, and provide as much detail as possible about nuances of the data, such as whether the code was considered the primary or secondary health end point, whether an emergency room visit led to hospital admission, and whether rates were age-adjusted.

To better characterize the pollutants being evaluated, the authors recommend including all time variables assessed (e.g., 8-hour, daily, annual), speciation of particulate matter, location of monitors, meas-urement method of the monitor instrument, and data on missing values and exceptional events. For statistical analyses, the recommendations include listing all uncertainty factors (e.g., *t*-statistics, *p*-value, 95% confidence interval, standard error of central estimate) and reporting null or statistically insignificant findings.

The authors say these commonsense ideas haven’t been adopted by epidemiologists for a variety of reasons, such as lack of awareness about their importance to risk assessors, unavailability of the information in the base data, or lack of space due to journal restrictions (although some journals now publish supplemental data at no additional cost). They suggest that workshops involving both epidemiologists and risk assessors would help get these recommendations out to their colleagues.

